# The Arclight Ophthalmoscope: A Reliable Low-Cost Alternative to the Standard Direct Ophthalmoscope

**DOI:** 10.1155/2015/743263

**Published:** 2015-09-17

**Authors:** James Lowe, Charles R. Cleland, Evarista Mgaya, Godfrey Furahini, Clare E. Gilbert, Matthew J. Burton, Heiko Philippin

**Affiliations:** ^1^Eastbourne District General Hospital, Kings Drive, Eastbourne BN21 2UD, UK; ^2^Kilimanjaro Christian Medical Centre Eye Department, P.O. Box 3010, Moshi, Tanzania; ^3^International Centre for Eye Health, London School of Hygiene & Tropical Medicine, Keppel Street, London WC1E 7HT, UK

## Abstract

*Background*. The Arclight ophthalmoscope is a low-cost alternative to standard direct ophthalmoscopes. This study compared the Arclight ophthalmoscope with the Heine K180 direct ophthalmoscope to evaluate its reliability in assessing the vertical cup disc ratio (VCDR) and its ease of use (EOU). *Methods*. Eight medical students used both the Arclight and the Heine ophthalmoscopes to examine the optic disc in 9 subjects. An EOU score was provided after every examination (a higher score indicating that the ophthalmoscope is easier to use). A consultant ophthalmologist provided the reference standard VCDR. *Results*. 288 examinations were performed. The number of examinations that yielded an estimation of the VCDR was significantly higher for the Arclight ophthalmoscope (125/144, 85%) compared to the Heine ophthalmoscope (88/144, 61%) (*p* < 0.001). The mean difference from the reference standard VCDR was similar for both instruments, with a mean of −0.078 (95% CI: −0.10 to −0.056) for the Arclight and −0.072 (95% CI: −0.097 to −0.046) for Heine (*p* = 0.69). The overall EOU score was significantly higher for the Arclight ophthalmoscope (*p* < 0.001). *Conclusion*. The Arclight ophthalmoscope performs as well as, and is easier to use than, a standard direct ophthalmoscope, suggesting it is a reliable, low-cost alternative.

## 1. Introduction

Vision 2020 is the global initiative, launched in 1999 by the World Health Organization (WHO) and the International Agency for the Prevention of Blindness (IAPB), with the aim of eliminating avoidable blindness. In the African region, WHO estimates that 26 million people are visually impaired and 6 million people are blind [1]. Significant progress has already been made in treating or preventing anterior segment eye diseases such as cataract and trachoma and an increasing proportion of the remaining burden of avoidable blindness in sub-Saharan Africa (SSA) is attributable to diseases of the back of the eye, particularly glaucoma and diabetic retinopathy [2].

The International Diabetes Federation predicts that the number of adults with diabetes in Africa will double from 12 million in 2010 to 24 million in 2030 [3]. Diabetic retinopathy is the commonest microvascular complication of diabetes [4] and, globally, is the leading cause of blindness in adults of working age [5]. The number of people in SSA with glaucoma was estimated to be 6.5 million in 2010 and is anticipated to increase to 8.4 million by 2020 [6]. Although no cure exists for glaucoma or diabetic retinopathy, early detection and timely management can help slow progression and improve prognosis [7, 8].

In many SSA countries the* per capita* number of ophthalmologists and the prevalence of blindness are inversely correlated; the former concentrated in major urban areas and the latter in poorer rural regions [9]. In addition to this, the overall numbers of eye health personnel are below that which is needed. The regional ratio of ophthalmologists in SSA is 2.3 per million population [10]. For example, in Malawi there are seven consultant ophthalmologists serving a population of 15.2 million [11]. Therefore, general healthcare workers, opticians, and allied eye care professionals often review patients with eye diseases. These groups need access to equipment and adequate training to allow them to examine and detect pathology in the posterior segment of the eye. However, standard direct ophthalmoscopes are expensive, ranging from USD $200 to 600 per instrument.

**Figure 1:**
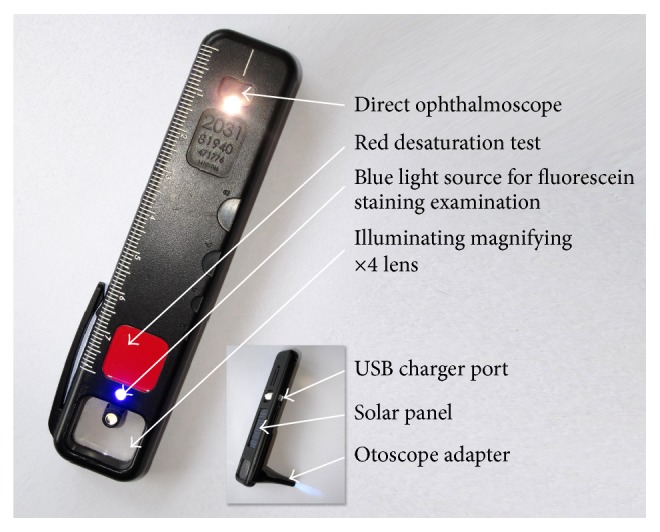
The Arclight direct ophthalmoscope with selected features highlighted.

The Arclight ophthalmoscope (http://www.arclightscope.com; Figure 1) provides a low-cost alternative to standard direct ophthalmoscopes. It is marketed at USD $7.50 when sold in bulk. It has a small direct ophthalmoscope at one end with an illuminating magnifying loupe (allowing examination of the anterior segment) and a detachable otoscope at the other end. It weighs 18 grams, uses three LED light sources, and has an inbuilt rechargeable battery powered by either an integrated solar panel (useful for mobile clinics in sub-Saharan Africa) or a USB port. The device has an adjustable lens slider with three different lenses, allowing for a rough correction of the patient's or examiner's refractive error. The device also incorporates a small colour vision test, a near visual acuity chart, a ruler, and a pupil size gauge.

The aim of this study was to assess if the Arclight ophthalmoscope performs as well as a conventional direct ophthalmoscope in the hands of final-year medical students (representative of nonspecialist users) in terms of estimating the vertical cup : disc ratio (VCDR) and its ease of use (EOU), in order to evaluate Arclight as an alternative to the standard direct ophthalmoscope. The VCDR was chosen as it is a clinically important measure in the assessment of glaucoma. The disc is a central and easily identifiable structure with sufficient variation in cup : disc ratios to provide numerical data for formal analysis [12, 13].

## 2. Materials and Methods

The study was approved by the Research Ethics Review Committee at Kilimanjaro Christian Medical University College (KCMUC), Moshi, Tanzania. It was conducted in the Department of Ophthalmology at Kilimanjaro Christian Medical Centre (KCMC). Written informed consent was provided by all participants.

In this study we compared the Arclight ophthalmoscope to the Heine K180 in terms of four measures: (1) accuracy of VCDR assessment, (2) ease of use (EOU) for the examiner using a score of 1–8 (Table 1), (3) the level of glare experienced by the subject using a score of 1–4 (Table 1), and (4) the perceived duration of the assessment using a score of 1–4 (Table 1) and scored by the subject as well. For measures (2) to (4) higher scores indicated better results.

Eight final-year medical students performed the examinations. To ensure a similar level of experience in ophthalmology, students who had taken more than one undergraduate course in ophthalmology were excluded. Students with a refractive error exceeding the corrective lenses of Arclight (−6 to +4) were also excluded. An introductory “refresher” session on direct ophthalmoscopy with particular reference to examination of the optic disc and assessment of the VCDR was provided. Following this, the examiners had a short practice session to familiarize themselves with both devices.

Nine healthy volunteers (18 eyes) acted as subjects and each was examined by the eight medical students. All subjects had one eye dilated at random using tropicamide 1% eye drops. Therefore, half of the examinations were performed on a dilated pupil. Each examiner assessed both eyes of each volunteer subject with both devices. They were randomly assigned to start with an Arclight or Heine ophthalmoscope. Examinations were conducted in two circuits. In the first circuit the examiners examined both eyes of each subject using either the Arclight or the Heine ophthalmoscope. In the second circuit the examiners changed ophthalmoscopes and reexamined both eyes of each subject. Within each circuit, all the right eyes were examined first, followed by all the left eyes. This was done as we recognized that there could be a tendency, because of the normal symmetry between eyes, not to grade the eyes in an independent manner [14]. A consultant ophthalmologist (HP), with a specialty interest in glaucoma, examined each subject with the Heine direct ophthalmoscope to provide the “reference standard” for VCDR measurement.

Data were recorded at the end of each examination by both the examiner and the subject. The examiner recorded the VCDR (range: 0.0 to 1.0) and an ease of use score (Table 1). The subject recorded the level of glare experienced and an impression of the length of the examination (Table 1).

Statistical analysis was performed using Student's* t*-test to compare differences between examiners' VCDR measurements and the reference standard. A Bland-Altman plot was calculated for both devices to provide a visual guide to the differences between examiners' VCDR scores and the “reference standard.” STATA version 13 was used to compute statistics and graphs. A chi-squared test was used to compare the proportion of examinations that yielded an estimation of the VCDR with the Arclight versus the Heine ophthalmoscope. Ease of use, “glare,” and “length of examination” were analyzed using chi-squared and Wilcoxon rank-sum tests.

## 3. Results and Discussion

### 3.1. Results

#### 3.1.1. VCDR Estimation

Each examiner performed 36 examinations (18 with the Arclight ophthalmoscope and 18 with the Heine ophthalmoscope, 9 dilated and 9 undilated eyes each), resulting in a total of 288 examinations. In total, 213 (74%) examinations resulted in an estimate of the VCDR. Significantly more of the Arclight examinations (125/144 [85%]) compared to the Heine ophthalmoscopes examinations (88/144 [61%]) produced a VCDR measure (*χ* ^2^, *p* < 0.001). For both devices significantly more VCDR estimates were possible through examination of dilated pupils: 71/125 (57%) for the Arclight (*χ* ^2^, *p* < 0.001) and 58/88 (66%) for the Heine (*χ* ^2^, *p* < 0.001).

**Figure 2:**
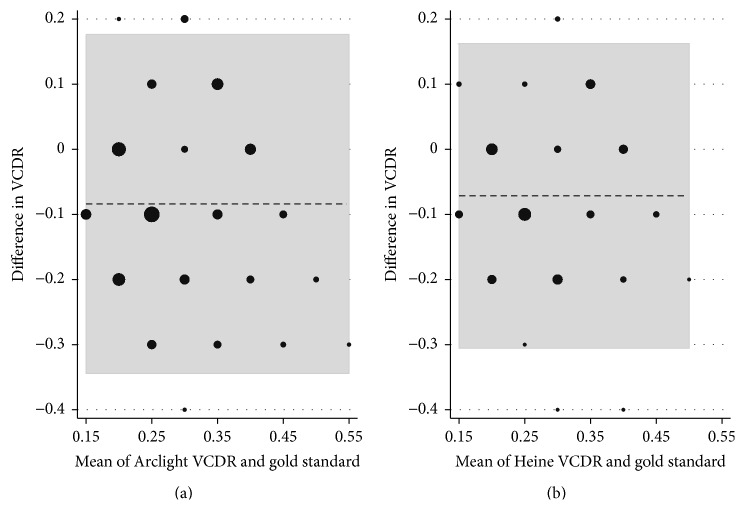
Bland-Altman plots showing the difference between the examiner's estimate of vertical cup : disc ratio (VCDR) and the reference standard, split by instrument. Plot (a) represents the Arclight direct ophthalmoscope and plot (b) represents the Heine K180 direct ophthalmoscope. Where there is exact agreement between the examiner and the reference standard the difference in VCDR is noted as 0. Any deviation from 0 represents underestimation (if negative) or overestimation (if positive) of the VCDR compared with the reference standard. The horizontal dotted line represents the mean of all observations (i.e., their mean deviation from the reference standard), and the grey area represents the proportion of all observations lying within 95% of the normal distribution for each of the two ophthalmoscopes. The size of each black dot is proportional to the number of observations it represents.

There was a very small difference between the reference standard VCDR measure and the Arclight measurements: mean difference −0.078 (95% CI: −0.10 to −0.056). There was a similar, very small difference for the comparison between the reference standard and Heine ophthalmoscope measurements: mean difference −0.072 (95% CI: −0.097 to −0.046). There was no difference in this mean performance between the two measures (*p* = 0.69). Bland-Altman plots were constructed for the difference in VCDR estimates between examiners and the reference standard (Figure 2).

Separate subanalyses of dilated and undilated pupils had similar results. For dilated pupils, the mean difference between the reference standard VCDR and the Arclight measurement was −0.087 (95% CI: −0.057 to −0.12) compared to −0.084 (95% CI: −0.053 to −0.12) for the Heine ophthalmoscope, with no difference between ophthalmoscopes (*p* = 0.9). For undilated pupils, the mean difference between the Arclight measurement and the reference standard was −0.067 (95% CI: −0.033 to −0.10) compared to −0.047 (95% CI: −0.004 to −0.089) for the Heine ophthalmoscope, with no difference between ophthalmoscopes (*p* = 0.47).

The total number of examinations that yielded a ≥0.2 difference in the VCDR from the reference standard was 37/125 (29.6%) and 25/88 (28.4%) for the Arclight and Heine ophthalmoscopes, respectively (*χ* ^2^, *p* = 0.85). A random effects model was performed which showed no significant between-cluster variation between the two eyes.

#### 3.1.2. Ease of Use

**Figure 3:**
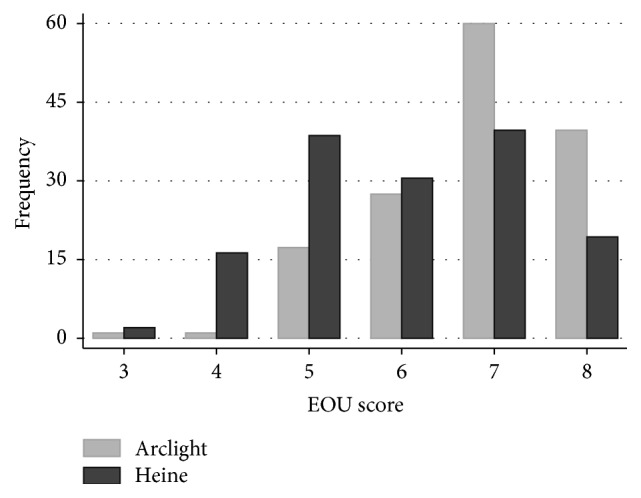
A histogram of the frequency of ease of use score for the Arclight and Heine direct ophthalmoscopes. 3: could identify red reflex. 4: could see vessels but not disc. 5: could identify disc but not vertical CD-ratio (VCDR). 6: could determine VCDR with a high level of difficulty. 7: could determine VCDR with a medium level of difficulty. 8: could determine VCDR with a low level of difficulty.

An ease of use score was obtained for all 288 examinations. The overall median score was significantly higher (Wilcoxon rank-sum, *p* < 0.001) for the Arclight (median 7, IQR 6 to 8) than the Heine (median 6, IQR 5 to 7), Figure 3. Both devices had higher EOU scores for dilated pupils: Arclight median score 7 (IQR 7-8) for dilated eyes versus 7 (IQR 5.75–7) for undilated pupils (Wilcoxon rank-sum, *p* < 0.001) and Heine median score 7 (IQR 6-7) versus 5 (IQR 5-6) (Wilcoxon rank-sum, *p* < 0.001) for dilated and undilated eyes, respectively. There were no significant differences in EOU scores between the first and second circuits.

#### 3.1.3. Examination Comfort

**Figure 4:**
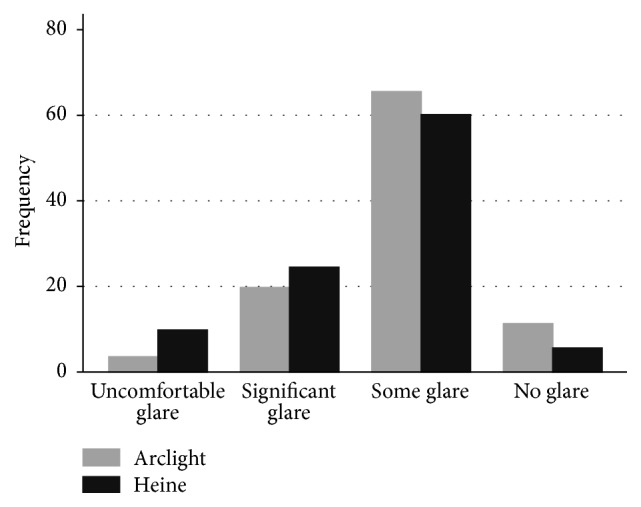
A histogram of the frequency of brightness reported by those examined for the Arclight and Heine direct ophthalmoscopes.

**Figure 5:**
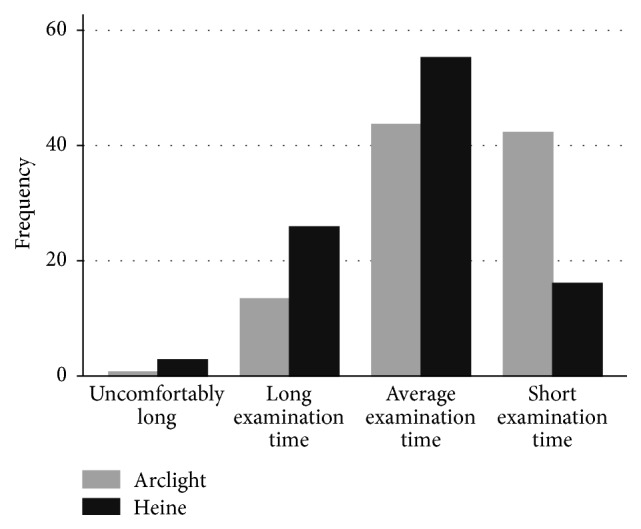
A histogram showing the subjective length of examination reported by those examined for the Arclight and Heine direct ophthalmoscopes.

Subject-rated “glare” and perceived “length of examination” data were obtained for 285/288 examinations. Responses were missing for three examinations. Participants reported significantly more “glare” from the Heine ophthalmoscope (*p* = 0.046): Arclight median score 3 (IQR 3-3), Heine median score 3 (IQR 2-3) (Figure 4). Similarly, participants reported significantly longer examinations for the Heine ophthalmoscope (*p* < 0.001): Arclight median score 3 (IQR 3-4), Heine median score 3 (IQR 2-3) (Figure 5). There were no significant differences in subject-rated “glare” between dilated and undilated pupils. However, subjects reported a significantly shorter “length of examination” when dilated pupils versus undilated pupils were examined (*p* < 0.001). This difference was also apparent when each device was compared separately.

### 3.2. Discussion

The Arclight ophthalmoscope aims to provide a reliable, low-cost alternative to the standard direct ophthalmoscope. We found no evidence of a difference between the Arclight ophthalmoscope and the Heine K180 direct ophthalmoscope in terms of accuracy of the VCDR measurement, performed by final-year medical students. However, the students found the Arclight significantly easier to use. We also found no clinically significant differences between the devices, with a similar proportion of examinations yielding a ≥0.2 difference in the VCDR compared to the reference standard for both the Arclight and Heine ophthalmoscopes.

Importantly, 85% of Arclight examinations yielded a VCDR estimation, compared to 61% with the Heine ophthalmoscope. This provides an additional indication that the Arclight ophthalmoscope is easier to use with added clinical benefits. From a patient perspective, the LED bulb in the Arclight ophthalmoscope resulted in a subjectively more comfortable examination, with significantly lower scores for both “glare” and “length of examination.”

The cost of an Arclight ophthalmoscope is considerably lower than Heine K180 direct ophthalmoscope or comparable instruments. For the current price of Heine direct ophthalmoscope (USD $365), one can buy 48 Arclight ophthalmoscopes at their marketed bulk order price (USD $7.5).

Arclight is the first direct ophthalmoscope to be specifically designed for low-income settings. However, it also has a potential application to training and education globally by providing a more affordable direct ophthalmoscope for students. In contrast to other low-cost direct ophthalmoscopes [13, 15], the Arclight has an adjustable lens power with three settings (+4, −3, and −6 dioptres). This simple adjustment will compensate for most patient and examiner refractive error. It also has an additional function as an otoscope in combination with the provided attachable device (Figure 1).

The human resources situation in SSA means that large numbers of patients are seen in rural areas or in mobile clinics by allied eye health professionals who often have limited access to equipment and training, making examination of the posterior segment of the eye impossible [10]. Due to its low cost, Arclight has the potential to be much more widely available, hence improving training opportunities and allowing examination of the optic disc more routinely. This will aid the early detection of glaucomatous disc changes. The earlier the glaucoma is detected and managed the better the prognosis with a reduced likelihood of progression to blindness is. The solar powered battery of the Arclight ophthalmoscope offers a further advantage for remote, rural clinics enabling the Arclight ophthalmoscope to be easily recharged in rural areas without access to power and also avoiding the expense of replacing batteries.

Although this study did not assess the accuracy of the Arclight ophthalmoscope in detecting abnormalities in the retina, it is possible that it could be used to detect diabetic retinopathy. The effective management of diabetic retinopathy in SSA will need a coordinated effort and faces multiple challenges. Burgess et al. highlighted a number of these including lack of training for opticians and other eye care workers in fundoscopy as well as poor equipment [11]. Arclight has the potential to help with both these challenges through greater access to direct ophthalmoscopes; however, this will need to be formally evaluated.

One of the limitations of our study was that all the eyes examined were healthy. It is therefore not possible to comment on how Arclight will perform in the presence of pathology. In everyday clinical practice it is clear that pathology such as cataract will be common, especially in low-income settings, and will generally make the examination of the posterior segment more difficult.

In conclusion, the Arclight ophthalmoscope provides a low-cost alternative to the standard direct ophthalmoscope. It is easier to use and more comfortable for the subject. It appears to provide comparable results when examining the VCDR. It has the potential to significantly improve access to equipment in low-income settings around the world. This could improve fundoscopy amongst eye care workers and enable routine examination of the posterior segment of the eye, which is an area that is becoming increasingly important in SSA. Further studies assessing the reliability of the Arclight ophthalmoscope in diabetic retinopathy detection and in the presence of other pathology would be useful.

## Figures and Tables

**Table 1:** Examination scales: (a) ease of use (examiner), (b) comfort scale (subject) and (c) length of examination (subject).

(a) Ease of use	(1) Could not use at all
	(2) Could not see the red reflex to even begin with
	(3) Could identify red reflex
	(4) Could see vessels but not disc
	(5) Could identify disc but not vertical CD-ratio (VCDR)
	(6) Could determine VCDR with a high level of difficulty
	(7) Could determine VCDR with a medium level of difficulty
	(8) Could determine VCDR with a low level of difficulty

(b) Comfort scale	(1) Uncomfortable glare
	(2) Significant glare
	(3) Some glare
	(4) No glare

(c) Length of examination	(1) Uncomfortably long
	(2) Long examination time
	(3) Average examination time
	(4) Short examination time

## References

[1] Pascolini D., Mariotti S. P. (2012). Global estimates of visual impairment: 2010. *British Journal of Ophthalmology*.

[2] Bastawrous A., Burgess P. I., Mahdi A. M., Kyari F., Burton M. J., Kuper H. (2014). Posterior segment eye disease in sub-Saharan Africa: review of recent population-based studies. *Tropical Medicine & International Health*.

[3] Shaw J. E., Sicree R. A., Zimmet P. Z. (2010). Global estimates of the prevalence of diabetes for 2010 and 2030. *Diabetes Research and Clinical Practice*.

[4] Sivaprasad S., Gupta B., Gulliford M. C. (2012). Ethnic variations in the prevalence of diabetic retinopathy in people with diabetes attending screening in the United Kingdom (DRIVE UK). *PLoS ONE*.

[5] Klein B. E. K. (2007). Overview of epidemiologic studies of diabetic retinopathy. *Ophthalmic Epidemiology*.

[6] Quigley H., Broman A. T. (2006). The number of people with glaucoma worldwide in 2010 and 2020. *British Journal of Ophthalmology*.

[7] Heijl A., Leske M. C., Bengtsson B., Hyman L., Bengtsson B., Hussein M. (2002). Reduction of intraocular pressure and glaucoma progression: results from the Early Manifest Glaucoma Trial. *Archives of Ophthalmology*.

[8] The Diabetes Control and Complications Trial Research Group (1993). The effect of intensive treatment of diabetes on the development and progression of long-term complications in insulin-dependent diabetes mellitus. *The New England Journal of Medicine*.

[9] Bastawrous A., Hennig B. D. (2012). The global inverse care law: a distorted map of blindness. *British Journal of Ophthalmology*.

[10] Palmer J. J., Chinanayi F., Gilbert A. (2014). Mapping human resources for eye health in 21 countries of sub-Saharan Africa: current progress towards VISION 2020. *Human Resources for Health*.

[11] Burgess P. I., Msukwa G., Beare N. A. V. (2013). Diabetic retinopathy in sub-Saharan Africa: meeting the challenges of an emerging epidemic. *BMC Medicine*.

[12] Mandal N., Harborne P., Bradley S. (2011). Comparison of two ophthalmoscopes for direct ophthalmoscopy. *Clinical and Experimental Ophthalmology*.

[13] McComiskie J. E., Greer R. M., Gole G. A. (2004). Panoptic versus conventional ophthalmoscope. *Clinical & Experimental Ophthalmology*.

[14] Li H., Healey P. R., Tariq Y. M., Teber E., Mitchell P. (2013). Symmetry of optic nerve head parameters measured by the heidelberg retina tomograph 3 in healthy eyes: the blue mountains eye study. *American Journal of Ophthalmology*.

[15] Armour R. H. (2000). Manufacture and use of home made ophthalmoscopes: a 150th anniversary tribute to Helmholtz. *British Medical Journal*.

